# Prevalence and Risk Factors of Cyberbullying and Its Association With Mental Health Among Adolescents in Saudi Arabia

**DOI:** 10.7759/cureus.32806

**Published:** 2022-12-21

**Authors:** Njoud Alrasheed, Sumaiya Nishat, Abdulelah Bin Shihah, Abdulaziz Alalwan, Hoda Jradi

**Affiliations:** 1 Department of Family and Community Medicine, King Saud University Medical City, Riyadh, SAU; 2 Department of Psychology, Middle East Technical University, Ankara, TUR; 3 Department of Family Medicine, King Faisal Specialist Hospital and Research Centre, Riyadh, SAU; 4 Department of Family and Community Medicine, King Saud Bin Abdulaziz University for Health Sciences College of Medicine, Riyadh, SAU; 5 Department of Public Health, King Saud Bin Abdulaziz University for Health Sciences College of Medicine, Riyadh, SAU

**Keywords:** traditional bullying, e-cigarettes, adolescents, mental-health, cyberbullying

## Abstract

Purpose

Advancements in internet technology are on the rise and so is the concern for its detrimental effects on youth like cyberbullying. Cyberbullying is on the rise and may cause adverse effects on mental health. The objective of the present study was to identify the prevalence of cyberbullying and its associated risk factors and to measure its association with mental health among adolescents.

Methods

An online self-administered questionnaire was distributed to 761 high school students aged 15 - 19 years from Riyadh, Saudi Arabia. A quantitative cross-sectional design was integrated, and logistic regression analysis was performed to determine the association. As part of assessing mental health, a questionnaire on the use of cigarettes, e-cigarettes, and hookah was also administered.

Results

The prevalence of cyberbullying was 18%. Although a significant association between cyberbullying and mental health status was obtained (OR = 1.04; p =0.03), the risk of Odds was found to be weak and therefore did not favor the hypothesis. The significant risk factors associated with cyberbullying include being traditionally bullied (OR= 4.76; *p* = <.001), e-cigarette use (OR = 2.73; *p* = <.001), and male gender (OR = 1.64; *p* = .04).

Conclusion

Despite the findings not favouring the hypothesis, a few striking associations were obtained in the study. Traditional bullying and e-cigarette use increased the risk of cyberbullying. This is a matter of rising concern since e-cigarette use has witnessed a surging rise in popularity. These findings may serve as early warning on the rising issue of cyberbullying and could pave way for formulating early preventive strategies and promulgate awareness by the concerned authorities.

## Introduction

With the evolution of the Internet in the late 20th century, the world has witnessed substantial advancements ranging from simple tasks like online shopping, and banking, extensive availability of social media applications, and communication platforms to more sophisticated use with Machine Learning and Artificial Intelligence. The advancement in technology has made the process of communication seamless, enabling connectivity with individuals across the globe within seconds. All these factors account for making the use of the internet a necessity rather than a luxury [1]. In this digital era, most teenagers have access to their own smart devices, accounting for up to one-third of global internet users under the age of 18 [2]. Although the internet is a boon in today’s digital era, it comes with a lot of disadvantages. This increased exposure has added to the likelihood of negative outcomes such as cyberbullying. The ever-increasing consumption of technology especially among adolescents is creating a surging concern globally [2].

Cyberbullying similar to traditional forms of bullying begins with a malicious intent to harm an individual repeatedly. Dehue et al. suggest three conditions that should satisfy for an act to be pronounced as cyberbullying - the attack must be intentional, occur repeatedly, and should cause psychological distress [3]. Cyberbullying occurs electronically through online platforms that offer messaging and calling services. The attack could be, written - via text messages, posting derogatory comments, visual - circulation of private or humiliating images, verbal - voice messages or calls, exclusion - deliberately excluding a participant from a conversation [4]. Moreover, revealing personal information without permission, blackmailing, threatening, and intimidating also include some ways through which cyberbullying could take place. Although both forms of bullying are abhorrent and are known to have a harmful impact on victims, cyberbullying is a growing issue of concern due to increased accessibility and extensive use of digital technology.

Adolescence is a period of critical development cognitively, socially, and morally. Research suggests that 50 percent of lifelong disorders mentioned in DSM-IV begin by the age of 14 and further increase to three-fourths by 24 [5]. The development of abstract thinking in this age leads to the enhanced thinking of self-concept. From a moral developmental perspective, adolescents are classified into a pre-conventional stage where their thinking revolves around just their own perceptions thereby neglecting those of others [6]. Therefore, a lack of complete development makes this age group more vulnerable to cyberbullying and susceptible to mental illnesses.

Although both cyberbullying and traditional bullying overlap in many aspects, it is the unique features of cyberbullying that set it apart [7]. One such instance is physical proximity. As the perpetrator and the victim do not belong in the same vicinity, the emotions and immediate reaction of the victim become unknown. This significantly decreases personal accountability and is referred to as the “disinhibition effect” [1]. Another such difference is the widespread availability and extent of global audiences. As geographical boundary limitations do not exist, an enormous number of spectators can be gathered, and the anonymity of the perpetrator makes it more widespread [6].

Adolescents involved in cyberbullying are highly prone to develop negative mental health and psychosocial issues. The effects are not limited to non-physical complications but can also be somatic [8]. Several studies have documented higher levels of anxiety, stress and depressive symptoms among cyberbullying victims [9-11]. Additionally, significant emotional problems, loss of confidence and self-destructive behaviors are observed. Physical complications include sleep problems, headaches, abdominal pain, loss of appetite and bed-wetting [8]. High correlation between suicide, risky behaviours such as substance abuse with cyberbullying has been demonstrated by various studies [12].

The rate of internet addiction and social media use is growing rapidly in Saudi Arabia especially among adolescents. The prevalence estimate has spiked from 4-6% in 2014-2015 to a whopping 30-60 % in less than half a decade in 2019 [13]. As of 2022, more than 98% of Saudi population are active internet users [14]. This significantly increases the risk of cyberbullying. A recent study done locally shows that 20.7% of youth are involved in cyberbullying [15]. Global prevalence estimates differ due to difference in measures and instruments. An extensive study involving 15,000 students in the United States found the prevalence rates to be 12% cybervictims, 4% cyberbullies and 3% cyberbully-victims [16]. Cyberbullying being a relatively new phenomenon, studies on the subject are limited and calls for more thorough investigation. The current study aimed to determine the prevalence of cyberbullying, to assess the correlation between cyberbullying and mental health among adolescents and to identify the risk factors associated with cyberbullying. It was hypothesized that there exists a positive association between cyberbullying and mental health among adolescents.

## Materials and methods

Participants and procedure

This quantitative observational cross-sectional study sample consisted of 761 high school students belonging to grades 10,11 and 12 from Riyadh, Saudi Arabia. The ages of the participants ranged from 15 to 19 years old. All participants with mental or learning disabilities that hindered their reading and comprehension abilities were excluded. 

A 95% confidence interval was chosen with a 5% margin of error. A required sample of 389 adolescents was obtained. To further the study, the number was multiplied by two to get a more reliable sample of a total of 778 adolescents. 

A pilot test was conducted to test the feasibility by administering to a group of 20 high school students of same age group but from different schools. This sample was not included in the final study sample. The pilot test reported no major difficulties. 

The reliability of the instrument was also checked by administering the instrument twice to the same group of high school students (n = 21) and within a one-week interval between the two administrations. There was a 95% agreement between the two administrations.

Measures 

Data were collected by an electronic method of survey using a self-administered questionnaire. The survey tool included three sections on demographic characteristics, mental health assessment, and questions related to smoking behavior. The socio-demographic assessment contained six questions on age, height, weight, and grade level (The high school years were categorized into three classes: first (Grade 10), second (Grade 11), and third (Grade 12), daily allowance, and family monthly income. Body mass index was derived by the height and weight recordings using the formula (weight in kilograms divided by height in meters squared). Mental health status was assessed using a valid and reliable Arabic Youth Mental Health questionnaire that had 21 questions related to emotional behavior like sadness, anxiousness, sleep disturbance, nervousness, feeling lonely, worried, having dizzy/lightheaded, emotionally drained, (headaches, stomach-aches, and nausea), thoughts of death and losing hope), followed by exposure to traditional bullying or e-bullying in the last one week. The total score for the mental health part of the questionnaire was computed by the summation of all 21 questions. 

The final section assessed the smoking status of the participant for using any type of tobacco (cigarettes, smokeless tobacco, and shisha/water pipe).

Data analysis 

Statistical Package for Social Sciences (IBM Corp. Released 2020. IBM SPSS Statistics for Windows, Version 27.0. Armonk, NY: IBM Corp) was utilized to analyze the data. Categorical variables were expressed as frequencies, percentages, means and standard deviations and Continuous variables were expressed as mean ± standard deviation. Logistic regression - univariate and multivariate was incorporated to obtain the main analysis.

Ethical considerations

The aims and objectives of the study were explained by the researcher. Care was taken to maintain confidentiality and anonymity. The right to withdraw from the study at any given point during the study was explained. As the study dealt with minor population, written consent from the parents was also taken. Permission was acquired from the parents and school officials prior to the distribution of the questionnaire. Ethical permission was obtained from the research ethics committee in the ministry of health (MOH) research unit (H-01-R-009)/Central IRB log 20-18E.

## Results

A total of 761 high school students aged between 15 to 19 years (mean age 17.5 years) comprising 400 (52.6%) females and 361 (47.4%) males were included in the study. The majority of the study participants were from the Third secondary grade (81.2%) and from high-income status greater than 12,000 SR (59.3%). Regarding the question on the perception of their own health, 60.1% opted for ‘excellent’ while none chose ‘poor’. The prevalence of cyberbullying in the present study was found to be 18% while traditional school bullying was 19.8%. As for the third domain of the survey that deals with smoking-related questions, a larger part of the population, did not report having used cigarettes (75.6%), e-cigarettes (77.8%), or hookah (81.2%). The total prevalence of cigarette and e-cigarette smoking was found to be 24.4% and 22.2% respectively. Among those who reported smoking, (20/186;10.7%) were daily cigarette smokers, (21/169;12.4%) were daily e-cigarette smokers while (30/143;21%) of hookah users preferred smoking one to two times a month. The other details related to smoking habits are displayed in Table 1.

**Table 1:** Sample Information (Cyberbullied Participants, N = 138)

Variable	Count(%) or M(SD)
Gender	
Female	49 (35.5%)
Male	89 (64.5%)
Grade	
First secondary	8 (5.8%)
Second secondary	14 (10.1%)
Third secondary	116 (84.1%)
Family income	
3,000 – 5,999 SR	22 (15.9%)
6,000 – 8,999 SR	13 (9.4%)
9,000 – 11,999 SR	20 (14.5%)
+12,000 SR	83 (60.1%)
Nationality	
Saudi	128 (92.8%)
Non-Saudi	10 (7.2%)
Age	17.57 (1.02)
BMI	23.73 (6.13)
Allowance	39.61 (86.55)
Total score – mental health	9.22 (5.08)
Perception of own health	
Poor	0 (0.0%)
Fair	0 (0.0%)
Good	12 (8.7%)
Very good	51 (37.0%)
Excellent	75 (54.3%)
Being bullied	
No	73 (52.9%)
Yes	65 (47.1%)
Having tried cigarettes	
No	75 (54.3%)
Yes	63 (45.7%)
Age of first smoking cigarettes	14.62 (2.59)
Days of smoking cigarettes during the last month	
0 days	117 (84.8%)
1 to 2 days	4 (2.9%)
3 to 5 days	4 (2.9%)
6 to 9 days	3 (2.2%)
10 to 19 days	4 (2.9%)
20 to 29 days	1 (0.7%)
Every day	5 (3.6%)
Cigarettes per day during the last month	
0	114 (82.6%)
<1	3 (2.2%)
1	6 (4.3%)
2-5	8 (5.8%)
6-10	3 (2.2%)
11-20	3 (2.2%)
+20	1 (0.7%)
Having tried e-cigarettes	
No	76 (55.1%)
Yes	62 (44.9%)
Days of smoking e-cigarettes during the last month	
0 days	110 (79.7%)
1 to 2 days	8 (5.8%)
3 to 5 days	5 (3.6%)
6 to 9 days	1 (0.7%)
10 to 19 days	4 (2.9%)
20 to 29 days	2 (1.4%)
Every day	8 (5.8%)
Source of e-cigarettes during the last month	
Gas station/convenience store	7 (5.1%)
Internet	10 (7.2%)
Someone bought it for me	3 (2.2%)
Borrowed	14 (10.1%)
Types of e-cigarettes used	
Rechargeable/refillable/tank type	34 (24.6%)
Throwable	4 (2.9%)
All types	10 (7.2%)
Reason for using e-cigarettes	
A friend or family member has used it	30 (21.7%)
Until I stop using other tobacco products	8 (5.8%)
Its cost is lower than other tobacco products	4 (2.9%)
It is easily obtained from other tobacco products	1 (0.7%)
Celebrities on TV or in movies use	0 (0.0%)
Because it is less harmful than other types of tobacco	9 (6.5%)
They are available in several flavors	5 (3.6%)
May be used in places where other tobacco products are not permitted	0 (0.0%)
I used it for another reason	4 (2.9%)
Having tried hookah/waterpipe	
No	93 (67.4%)
Yes	45 (32.6%)
Age of first using hookah/waterpipe	15.46 (2.39)
Days of using hookah/waterpipe during the last month	
0 days	118 (85.5%)
1 to 2 days	9 (6.5%)
3 to 5 days	2 (1.4%)
6 to 9 days	3 (2.2%)
10 to 19 days	1 (0.7%)
20 to 29 days	2 (1.4%)
Every day	3 (2.2%)
Location of using hookah/waterpipe during the last month	
My house	12 (8.7%)
Friend’s house	5 (3.6%)
Family member’s house	0 (0.0%)
Hookah bar	4 (2.9%)
Café/restaurant	2 (1.4%)
Another place	3 (2.2%)
Source of tobacco products during the last month	
Gas station/convenience store	13 (9.4%)
Internet	5 (3.6%)
Someone bought it for me	1 (0.7%)
Borrowed	2 (1.4%)
Family member	2 (1.4%)
Friend	5 (3.6%)
Thoughts of quitting	
During the next 30 days	9 (6.5%)
During the next 6 months	2 (1.4%)
During the next 12 months	5 (3.6%)
Yes, but not during the next 12 months	6 (4.3%)
No	11 (8.0%)

The risk factors of cyberbullying by univariate analysis are shown in Table 2. Males, higher mental health scores, those who were bullied, having tried any form of smoking - cigarette, e-cigarette or hookah were associated with a significant risk of cyberbullying. Furthermore, the Odds of risk associated with cyberbullying was highest among those who were bullied (OR 5.5; p <0.001) followed by e-cigarette users (OR 4; p <0.001), cigarette users (OR 3.4; p <0.001) and hookah smokers (OR 2.6; p <0.001). In addition, being a male increased the Odds by 2.3 times for cyberbullying.

**Table 2:** Risk Factors of Cyberbullying – Univariate Logistic Regression *Note.* Reference levels for categorical variables are in brackets.

Variable	OR	95% CI	p-value
Demographics			
Gender (female)	2.34	1.60-3.44	< .001
Age	1.05	0.88-1.26	.606
BMI	1.02	0.98-1.05	.325
Class (first grade)			.518
Second grade	0.71	0.28-1.85	.485
Third grade	1.01	0.46-2.24	.979
Family income (3,000-5,999 SR)			.883
6,000-8,999 SR	0.77	0.36-1.63	.486
9,000-11,999 SR	0.81	0.41-1.58	.533
+12,000 SR	0.90	0.53-1.52	.700
Allowance	1.00	1.00-1.00	.516
Nationality (Non-Saudi)	0.63	0.30-1.32	.215
Health			
Total score – mental health	1.07	1.03-1.10	< .001
Perception of own health	0.87	0.67-1.14	.313
Being bullied (No)	5.56	3.71-8.33	< .001
Tobacco products usage			
Having tried cigarettes (No)	3.42	2.32-5.04	< .001
Age of having first tried cigarettes	0.96	0.85-1.08	.491
Cigarettes smoked per day – last month	1.24	1.07-1.45	.005
Having tried e-cigarettes (No)	3.93	2.65-5.84	< .001
Having tried a hookah/waterpipe (No)	2.59	1.71-3.93	< .001
Age of having first tried a hookah/waterpipe	1.01	0.88-1.17	.870

Further analysis by Multivariate Logistic Regression showed the stronger risk factors that remained significantly associated with cyberbullying as shown in Table 3. The likelihood of being cyberbullied was found to be significantly higher for those who were bullied (OR 4.76 : p <0.001), e-cigarettes users (OR 2.73; p < 0.001), Males (OR 1.6: p = .04) and higher mental health scores (OR=1.04; p = 0.03) remain weakly associated. However, hookah and cigarette use did not remain significant in the analysis.

**Table 3:** Multivariate Logistic Regression Note: Reference levels for categorical variables are in brackets.

Variable	Log OR	SE	OR	95% CI	p-value
Intercept	-3.00	0.28	0.05	-	< .001
Gender (female)	0.49	0.24	1.64	1.02-2.63	.040
Total score – mental health	0.05	0.02	1.05	1.01-1.09	.030
Being bullied (No)	1.56	0.23	4.76	3.06-7.40	< .001
Having tried cigarettes (No)	0.45	0.31	1.56	0.86-2.84	.146
Cigarettes smoked per day – last month	0.01	0.10	1.01	0.82-1.23	.962
Having tried e-cigarettes (No)	1.01	0.30	2.73	1.52-4.93	< .001
Having tried a hookah/waterpipe (No)	-0.25	0.32	0.78	0.41-1.48	.449

## Discussion

The present study investigated the prevalence and risk factors of cyberbullying and determined the correlation between cyberbullying and mental health among adolescents. It was hypothesized to have a positive association between cyberbullying and mental health among adolescents. The study found no significant association between the two variables. Although the hypothesis was not supported, a striking significance was found between cyberbullying and the use of e-cigarettes. Furthermore, a strong correlation was found between having experienced a traditional form of bullying which increased the odds by 4.76 times, and male predominance 

The strength of the association between cyberbullying and mental health has widely been tested and demonstrated. Although the association was not supported (OR = 1.05) in the present study, there exists wide literature that strongly supports the association. Therefore, the results of this study serve as a means to predict upcoming trends in the region. A larger population-based study with a longitudinal design is highly recommended. The detrimental consequences of cyberbullying on mental health may include feelings like depression, anxiety, nervousness, and loneliness. Furthermore, somatic symptoms include sleeplessness, headaches, nausea, and stomach aches. Cyberbullied victims often feel isolated, dehumanized, and helpless. Such tendencies escalate the possibility of engaging in substance abuse, self-injurious behavior, and even suicidal thoughts [17]. According to the report released by World Health Organization in 2022, suicide is the second leading cause of death only after accidents and injuries among 15 -19 years age group [18]. These consequences of cyberbullying necessitate the formulation of urgent preventive measures. 

The association of e-cigarette use as a risk factor for cyberbullying is an important finding of this study. In line with our findings, a longitudinal study done in the United States assessing 2,768 adolescents reported a proportional rise in cyberbullying among substance users including e-cigarettes [19]. The use of e-cigarettes has gained immense popularity and has witnessed rapid growth due to their ease of availability and the option to recharge or simply refill the tank makes it more appealing to the youth [20]. In addition, being a relatively healthier variant compared to conventional cigarettes makes it a preferred option [21]. Studies have related its use to adverse mental health effects like depression [21]. In accordance with our findings, another recent study from Germany indicated an empirical relationship between cyberbullying and e-cigarette use among students [22]. However, research in this field is in a nascent stage and enormous scope exists for in-depth qualitative and quantitative studies relating to the use of e-cigarettes and their impact on mental health behavior and social media use.

The prediction of gender dominance in cyberbullying has been widely inconsistent throughout the literature. A study done by assessing 150 Arab American population found male adolescents to be significantly associated with cyberbullying concurring with our study [8]. On the contrary, several studies also found female adolescents to be more significant in cyberbullying victimization than males of the same age [9,10,23]. One reason for this inconsistency could be explained by the difference in social construction and cultural variances among societies. Research also suggests that females spend greater amounts of time social networking which increases the likelihood of getting cyberbullied. While males on the other hand, largely get involved in gaming thereby reducing their time networking [24]. Moreover, there are several other studies that demonstrate no gender differences for cyberbullying [25-26].

In line with the existing research, a significant association between traditional forms of bullying and cyberbullying was found. Prevalence of physical bullying and cyberbullying was found to be 19.8% and 18.1% respectively. Based on natural dispositional characteristics of females, it can be said that females are likely to engage in indirect bullying such as spreading rumors in contrast to male’s propensity to engage in more direct actions such as hitting. Such inherent attributes translate to cyberbullying as a more suitable medium for females and physical bullying more suitable for males [17].

There are a few limitations that should be considered while interpreting the findings of this study. To better assess the directionality of the association, a longitudinal study design is required. Causal relationship between cyberbullying and mental health effect cannot be understood by cross-sectional study design as performed in this study. The questionnaire used to assess the mental health of the participants questioned details only about the past one week. This excludes participants who experienced symptoms prior to the given period. Moreover, the symptoms experienced were not cross-checked with professionals. Therefore, this could question the reliability. In addition, alternative methods of assessment such as reports from parents, friends and peers were not used. This would further enhance the reliability of the data.

Despite these limitations, the study provides important findings since there is paucity of literature in this topic from the Saudi Arabian region. This calls for widespread research on the topic using longitudinal study designs and incorporating more diverse population and associated variables. This would help evaluate the strength of association over a period of time. Moreover, it can be made more specific to examine and analyze risk factors associated with it. These findings advocate for an implementation of awareness programs among parents and educational institutions. School students should be educated on this topic by integrating it into school curriculum from an early age. Young children should be made familiarized with the cause and effect of cyberbullying and in case of any such encounter, must be guided to take the right steps.

## Conclusions

Adolescents’ exposure to cyberbullying is on the rise. Although a significant impact on mental health was not found among Saudi Arabian population, new prominent trends such as e-cigarette use associated with cyberbullying have been identified. Recognising such predictive trends helps control burgeoning increase by incorporating early preventive measures. 
